# Test–Retest Reliability and Reliable Change Estimates for Four Mobile Cognitive Tests Administered Virtually in Community-Dwelling Adults

**DOI:** 10.3389/fpsyg.2021.734947

**Published:** 2021-10-21

**Authors:** Ryan Van Patten, Grant L. Iverson, Mélissa A. Muzeau, Heidi A. VanRavenhorst-Bell

**Affiliations:** ^1^Department of Physical Medicine and Rehabilitation, Harvard Medical School, Boston, MA, United States; ^2^Spaulding Rehabilitation Hospital, Charlestown, MA, United States; ^3^Home Base, A Red Sox Foundation and Massachusetts General Hospital Program, Charlestown, MA, United States; ^4^Sports Concussion Program, MassGeneral Hospital for Children, Boston, MA, United States; ^5^Spaulding Rehabilitation Institute, Charlestown, MA, United States; ^6^Sporttesting, Grenoble, France; ^7^Department of Human Performance Studies, Wichita State University, Wichita, KS, United States

**Keywords:** Mobile cognitive testing, ecological momentary assessment, technology, psychometrics, assessment, reaction time, attention, working memory

## Abstract

**Objective**: Remote mobile cognitive testing (MCT) is an expanding area of research, but psychometric data supporting these measures are limited. We provide preliminary data on test–retest reliability and reliable change estimates in four MCTs from SWAY Medical, Inc.

**Methods:** Fifty-five adults from the U.S. Midwest completed the MCTs remotely on their personal mobile devices once per week for 3 consecutive weeks, while being supervised with a video-based virtual connection. The cognitive assessment measured simple reaction time (“Reaction Time”), go/no-go response inhibition (“Impulse Control”), timed visual processing (“Inspection Time”), and working memory (“Working Memory”). For each cognitive test except Working Memory, we analyzed both millisecond (ms) responses and an overall SWAY composite score.

**Results:** The mean age of the sample was 26.69years (SD=9.89; range=18–58). Of the 55 adults, 38 (69.1%) were women and 49 (89.1%) used an iPhone. Friedman’s ANOVAs examining differences across testing sessions were nonsignificant (*p*s>0.31). Intraclass correlations for Weeks 1–3 were: Reaction Time (ms): 0.83, Reaction Time (SWAY): 0.83, Impulse Control (ms): 0.68, Impulse Control (SWAY): 0.80, Inspection Time (ms): 0.75, Inspection Time (SWAY): 0.75, and Working Memory (SWAY): 0.88. Intraclass correlations for Weeks 1–2 were: Reaction Time (ms): 0.75, Reaction Time (SWAY): 0.74, Impulse Control (ms): 0.60, Impulse Control (SWAY): 0.76, Inspection Time (ms): 0.79, Inspection Time (SWAY): 0.79, and Working Memory (SWAY): 0.83. Natural distributions of difference scores were calculated and reliable change estimates are presented for 70, 80, and 90% CIs.

**Conclusion**: Test–retest reliability was adequate or better for the MCTs in this virtual remote testing study. Reliable change estimates allow for the determination of whether a particular level of improvement or decline in performance is within the range of probable measurement error. Additional reliability and validity data are needed in other age groups.

## Introduction

Mobile cognitive testing (MCT) – brief, repeated cognitive tests delivered through mobile devices – is of considerable interest to the neuropsychology community. Its rapidly growing popularity is due in part to the downstream effects of the global COVID-19 pandemic (i.e., physical distancing), coupled with the increasing availability of wireless networks (Internet World Stats – Usage and Population Statistics, 2020) and smart phones (Pew Research Center, 2019). MCTs have a number of advantages over traditional neuropsychological testing, including remote, automated administration and scoring, sensitivity to fluctuating physiological states (e.g., arousal and mood), and the potential for improved ecological validity (Allard et al., 2014; Moore et al., 2017a; Sliwinski et al., 2018; Koo and Vizer, 2019; Weizenbaum et al., 2020). Moreover, due to the ease of repeated testing, MCT data are frequently aggregated, thereby enhancing stability in the estimation of cognitive functioning (Allard et al., 2014; Sliwinski et al., 2018). In other words, MCTs could allow for a repeatable, dynamic, real-world assessment of cognitive functioning, which has the potential for benefits in a wide variety of healthy and clinical populations, given the importance of understanding cognitive functioning outside of controlled clinical environments. However, MCTs are intended as an adjunct to rather than a replacement of traditional neuropsychological testing, which has several advantages, including the precise control of an examinee’s environment, an in-depth assessment of multiple cognitive domains, and a variety of available tests with large normative datasets.

Despite the need for both physically distant cognitive assessment and brief, repeatable, automated tests, a recent systematic search of available MCTs reported that only seven out of 25 included any psychometric data, with only one out of 25 having extensive supporting data (i.e., norms, reliability, validity, sensitivity, and specificity; Charalambous et al., 2020). For clinical scientists to begin using MCTs in neuropsychological research, rigorous psychometric evaluations of the tests must first be conducted.

SWAY Medical, Inc., offers an app that includes a suite of four MCTs assessing reaction time, impulse control, timed visual processing, and working memory. Data are measured *via* touch screen as well as tri-axial accelerometry (i.e., motion detection), which can reduce latencies from 50–200 ms (in conventional touch-screens) down to 1–2ms (Plant and Quinlan, 2013; Patterson et al., 2014; Amick et al., 2015; Woods et al., 2015; Burghart et al., 2019; SWAY Medical, LLC., 2020; VanRavenhorst-Bell et al., 2021). Therefore, SWAY MCTs might have an advantage over other mobile tests due to less variation in response time measurements (i.e., error variance) across devices and operating systems. However, little psychometric evidence is currently available for these tests. Burghart et al. (2019) reported good test–retest reliability data for the reaction time test, but the other three SWAY MCTs were not included in their study. They also reported a significant correlation (*r*=0.59) between the SWAY reaction time measure and a validated desktop-based test of reaction time. VanRavenhorst-Bell et al. (2021) investigated psychometric properties of the SWAY reaction time, timed visual processing, and impulse control tests in a sample of 88 healthy adults (aged 18–48). The authors reported preliminary evidence pertaining to convergent and discriminant validity of the SWAY tests when correlated with the ImPACT Quick Test. Half (12 of 24) of the bivariate correlation coefficients were statistically significant, with *r* values ranging from 0.22 to −0.46.

The prior studies discussed above administered SWAY tests in person, and neither reported on estimates of reliable change. The purpose of the current study is to examine test–retest reliability and reliable change estimates in the four SWAY MCTs, administered remotely in a sample of community-dwelling adults. We hypothesized that test–retest reliability estimates would be at least adequate in all four MCTs.

## Materials and Methods

### Participants

Participants were 61 adults, aged 18 and older, recruited through print materials and technology-based communications dispersed across a university and the U.S. Midwest I-35 corridor. The current study data were collected together with SWAY balance test data (to be presented in a separate paper). The study was conducted during the COVID-19 pandemic. The original recruitment goal was to enroll at least 50 participants, and as many as 100. After recruiting 61 people, data collection was discontinued due to study personnel limitations.

Exclusion criteria were the following self-reported medical conditions, assessed with the use of the *Physical Activity Readiness Questionnaire Plus*: musculoskeletal injury impacting movement/balance, neurological dysfunction, uncorrected vision, or a vestibular condition. Participants were also excluded if they were unable to maintain a videoconferencing connection during the testing sessions, or if they did not have a smart device capable of downloading and running the SWAY application. Of the 61 original participants, one withdrew from the study due to unforeseen medical issues, one withdrew due a time commitment, and four were removed due to equipment failure that prevented their data from being recorded. The final sample included 55 adults. All participants provided informed consent to participate and the study procedures were approved by the affiliate university’s Institutional Review Board.

### Materials/Procedures

The four SWAY tests were administered remotely, on participants’ personal mobile devices. The SWAY tests can be used on any device with an iOS version of 9.3 or higher and an Android version 7.0 or higher. A prior study showed that, within these constraints, the SWAY application can be administered on different mobile devices and operating systems without affecting measured data (VanRavenhorst-Bell et al., 2021). In order to improve adherence to the study protocol, all sessions were supervised by a research assistant who connected to the participant using video-based virtual connections.

Participants completed the four SWAY MCTs twice per week for 3 consecutive weeks. Week 1 (but not Week 2 or Week 3) also included an unscored practice administration for all four MCTs, in order to allow for familiarization. This led to three scores for each test in Week 1 (two of which were retained for data analysis), two scores for each test in Week 2, and two scores for each test in Week 3. The two test administrations within each week were averaged to create a mean score for each of the four tests, which allows for more stable estimates of cognitive functioning (see, e.g., Allard et al., 2014; Lange and Süß, 2014; Moore et al., 2017b; Sliwinski et al., 2018). The one-week test–retest interval is consistent with reliability studies of the SWAY balance tests (Amick et al., 2015).

The procedure described above matches recommendations for administration of SWAY MCTs. With longer test–retest intervals (e.g., 1–2months or more), it is typically recommended that examiners administer a practice test before each administration. In the current study, we elected not to include practice tests in Weeks 2 and 3 due to the short test–retest interval.

The SWAY protocol consisted of four MCTs. For the Simple Reaction Time test, the examinee holds their mobile device horizontally (landscape) and moves the device as rapidly as possible in any direction when the screen color changes from white to orange. The test starts after a variable delay of 2–4s in order to prevent the examinee from anticipating the stimulus ahead of time. The examinee completes five total trials. The most rapid and the slowest trial reaction times are both excluded in order to remove outliers and better capture the examinee’s typical response times. Following those exclusions, the values from the three remaining trials are averaged to calculate the score for the test.

For the Impulse Control (go/no-go) test, the examinee again holds their device horizontally and then moves it as rapidly as possible in any direction when a green circle with a white check mark is displayed on a blank screen. They do not move the device if a red circle with a white “*X*” is presented on a blank screen. The test begins after a variable delay of 2–4s. Eight total trials are administered (five “go” trials and three “no-go” trials). The five “go” trials are retained for scoring. Of these five trials, the most rapid and the slowest reaction times are both excluded and the values from the three remaining trials are averaged to calculate the score for the test.

**Figure 1:**
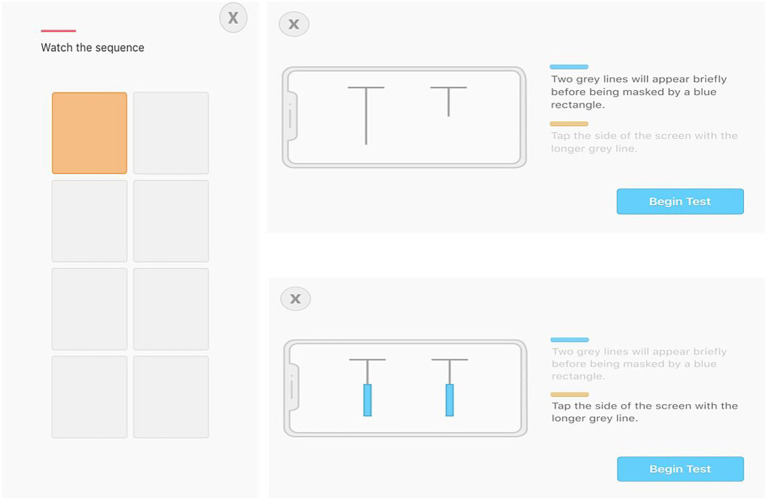
Screenshots of Working Memory **(left)** and Inspection Time **(right)** tests.

During the Inspection Time test, examinees hold their device horizontally. They see two T-shaped lines, one on each side of the screen. One of the two lines is long and one is short. The long end of two “Ts” is quickly hidden and the examinee taps the device screen on the side where the longer line was presented. They do not tap the device screen if they are unsure about which of the two lines is longer (see Figure 1). The test begins after a variable delay of 1–2s. The display interval begins at ~102ms and reduces by 1 screen refresh (~17ms) for each correct response until the user reaches 1 screen refresh. An additional trial is completed at 17ms to verify the score and the test is completed. When an incorrect answer occurs, 1 screen refresh (~17ms) is added to the next trial until a correct response is recorded. After an incorrect response, the examinee must earn two correct responses at a given interval before reducing by 1 screen refresh again. If an examinee gets every trial correct, including two trials at 1 screen refresh, they have completed the test. If the examinee makes two incorrect responses at any refresh interval, they must repeat and get two in a row correct at that interval with one more screen refresh to complete the test. The maximum number of trials is 20. An examinee’s score is the screen refresh rate at the conclusion of the test.

Finally, for the Working Memory test, examinees hold the device vertically. They first see three letters (all consonants) on the screen for 3s and then are asked to remember the letters. Next, the letters disappear and a 2 (columns)×4 (rows) grid of squares appears. One of the squares briefly flashes orange and then the examinee touches the square that flashed orange. Two squares then turn orange, one at a time, and then the examinee reproduces the sequence on the grid. The sequence continues to lengthen until the examinee makes one mistake; at that point, the grid disappears, they type in the three letters shown at the beginning of the test, and the test concludes (see Figure 1). The score for this test is created with a formula that accounts for both accurate recall of the three letters and progress through the grid sequence. The Working Memory SWAY Score is calculated in two steps. First, the maximum sequence length achieved is assigned a SWAY score, as follows: 0=0, 1=25, 2=50, 3=64, 4=67, 5=70, 6=73, 7=76, 8=79, 9=82, 10=85, 11=88, 12=91, 13=94, 14=97, and≥15=100. Second, three points are subtracted for each consonant that is incorrectly recalled. For example, a Sequence Length of 6, with three out of three consonants correctly recalled would be a SWAY score of 73. A Sequence Length of 4, with one out of three consonants correctly recalled would be a SWAY score of 61 (67 – 3 – 3).

The Reaction Time, Impulse Control, and Inspection Time tests all consist of two indices: (a) millisecond (ms) reaction times, and (b) an overall SWAY score. The Working Memory test does not have a reaction time component and is summarized with a single SWAY score.

### Statistical Analyses

Descriptive statistics for continuous variables are presented as the mean, standard deviation (SD), median (Md), range, and interquartile range (IQR); categorical variables include the sample size for each variable (*n*) and the proportion of the overall sample (%). We examined distributional characteristics of the four SWAY tests through a visual inspection of the histograms and skewness/kurtosis statistics. Several SWAY variables were not normally distributed, so nonparametric statistics are presented where appropriate.

We report intraclass correlation coefficients (ICCs) as an estimate of test–retest reliability. Interclass correlations such as Pearson’s and Spearman’s *r* measure relationships between variables in different classes of measurement. That is, the Pearson product-moment correlation is used to assess the strength and direction of the linear relationship between two variables, and the Spearman rank-order correlation is used to measure the strength and direction of the monotonic relationship between two variables. Conceptually, the ICC is often recommended over Pearson or Spearman correlations to evaluate test–retest reliability because test–retest reliability examines two or more scores within the same class of measurement (Kroll, 1962; McGraw and Wong, 1996; Bédard et al., 2000; Weir, 2005; Koo and Li, 2016). ICC values can be interpreted as the proportion of variance in observed scores that can be ascribed to true score variance. In other words, if the ICC is 0.80, then 80% of the observed score variance results from true score variance and 20% results from error.

The first step in assessing test–retest reliability using the ICC is to test for systematic error (e.g., practice effects) using a repeated measures analysis; we did so using Friedman’s ANOVAs. Next, we calculated ICCs using a mean-rating, absolute-agreement, two-way mixed effects model, which is appropriate for test–retest reliability where multiple measurements are averaged to produce a composite score (Shrout and Fleiss, 1979; McGraw and Wong, 1996; Field, 2005; Weir, 2005; Koo and Li, 2016). We used data from Weeks 1, 2, and 3 to calculate ICCs for test–retest reliability.

Following the assessment of test–retest reliability, we calculated the natural distribution of the difference scores for Week 2 minus Week 1. For example, a 10% difference score for Week 2 minus Week 1 refers to the score that occurs in ≤10% of the full sample. These data are presented in order to examine how closely the calculated reliable change estimates align with the actual distribution of difference scores.

A reliable change methodology was used to estimate measurement error surrounding the test–retest difference scores. The “Reliable Change Index” was originally proposed by Jacobson and Truax (1992) and a number of authors proposed modifications and refinements over the years (Chelune et al., 1993; Speer and Greenbaum, 1995; Hsu, 1999; Iverson, 2001). In order to define reliable change for clinical interpretation, we used two-tailed 70, 80, and 90% CIs (*z*=1.04, 1.28, and 1.64, respectively). The values produced provide the reader with the number of change points (increase or decrease from Week 1 to Week 2) necessary to reach the threshold of a reliable change at three different levels of confidence. The formulas are provided below and can be used to calculate reliable change for any desired CI. The calculation requires knowledge of the SD from test and retest, as well as the test–retest correlation coefficient. In this case, we used ICCs from Weeks 1 and 2 (not Week 3) as test–retest coefficients in order to reflect reliable change estimates from two time points. The steps for calculating the SE_diff_ are listed below.

1. SEM_1_= $SD\sqrt{1-r_{12}}$ Standard deviation from time 1 multiplied by the square root of 1 minus the test–retest coefficient (ICC).
2. SEM_2_= $SD\sqrt{1-r_{12}}$ Standard deviation from time 2 multiplied by the square root of 1 minus the test–retest coefficient (ICC).
3. SE_diff_= $\sqrt{SEM_{1}^{2}+SEM_{2}^{2}}$ Square root of the sum of the squared SEMs for each testing occasion.
4. Reliable Change CIs=The SE_diff_ is multiplied by the following z scores: ±1.04 (70% CI), ±1.28 (80% CI), and ±1.64 (90% CI).

The reliable change method used in the current study has a similar purpose to the minimal detectable change (MDC; Stratford et al., 1996) and the minimal clinically important difference – identifying clinically meaningful change over time. Both the current reliable change method and the MDC rely on the SE of measurement in their calculation. However, unlike the current reliable change approach, the MDC does not use the SE of the difference score in its calculation: 90% MDC=1.64×SEM×√2.

Statistical significance was set *a priori* at *p*<0.05. All analytic procedures, with the exception of the reliable change calculations, were carried out in IBM SPSS, Version 27.0.

## Results

**Figure 2:**
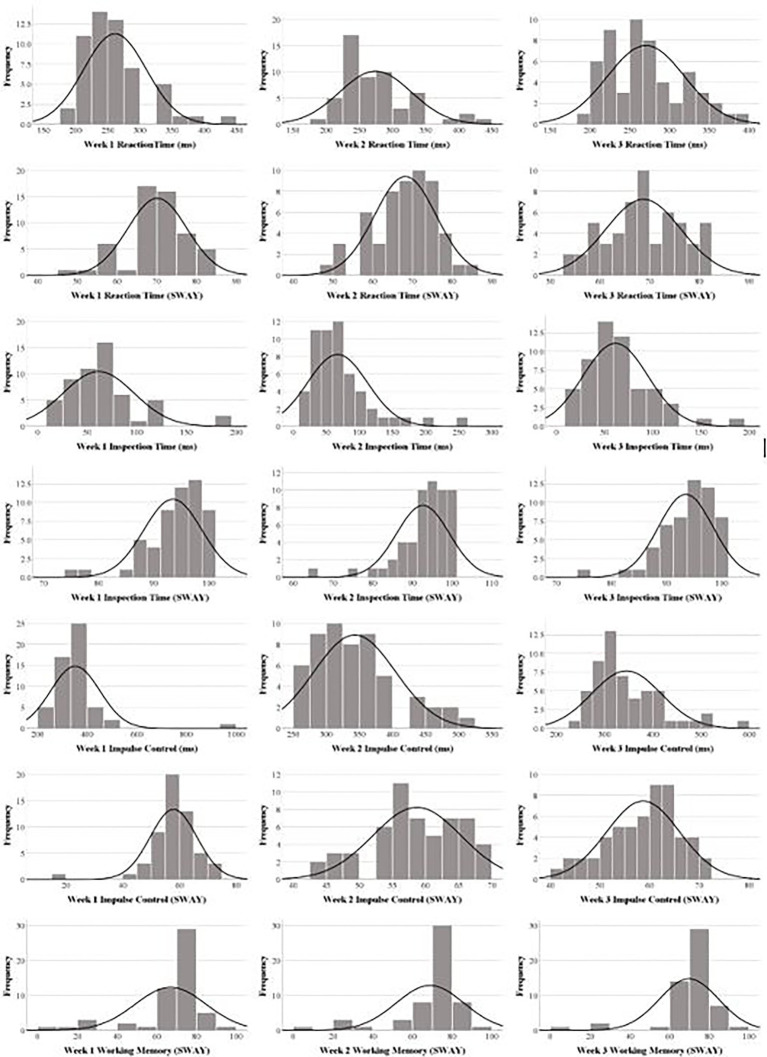
Histograms for the four SWAY tests.

The mean age of the sample was 26.69years (SD=9.89; Md=23.00; range=18–58; IQR=20–30). Of the 55 adults, 38 (69.10%) were women and 49 (89.09%) used an iPhone for the testing (the remaining six reported using phones with an Android operating system). Descriptive data for the five tests and indices are presented in Table 1. Histograms for each test at each time interval are presented in Figure 2. Friedman’s ANOVAs were all nonsignificant (*p* values, range=0.32–0.62; Table 2), suggesting that test scores did not differ across Weeks 1, 2, and 3. As seen in Table 3, test–retest ICCs for Weeks 1–3 ranged from 0.68 (Impulse Control, ms) to 0.88 (Working Memory), and ICCs for Weeks 1–2 ranged from 0.60 (Impulse Control) to 0.83 (Working Memory).

**Table 1:** Descriptive statistics for the SWAY mobile cognitive tests.

	*N*	Mean	*SD*	Md	IQR	Range
**Week 1**						
Reaction Time (ms)	55	261.19	48.79	252.50	229.00–277.50	195.50–427.50
Reaction Time (SWAY)	55	70.16	7.47	70.60	66.99–74.97	48.86–82.65
Impulse Control (ms)	55	352.08	98.84	339.50	309.00–366.00	241.00–961.50
Impulse Control (SWAY)	55	57.93	8.23	58.37	54.96–61.77	18.29–72.56
Inspection Time (ms)	55	61.51	35.76	59.50	34.00–76.50	17.00–187.00
Inspection Time (SWAY)	55	93.45	5.26	93.75	91.25–97.50	75.00–100.00
Working Memory (SWAY)	55	66.97	17.84	71.50	67.00–76.00	0.00–91.00
**Week 2**						
Reaction Time (ms)	55	274.33	54.17	262.50	233.50–296.50	184.50–426.00
Reaction Time (SWAY)	55	68.24	7.76	69.26	64.20–74.09	49.81–85.51
Impulse Control (ms)	55	343.08	61.62	332.50	292.50–372.50	258.50–516.00
Impulse Control (SWAY)	55	58.69	6.66	59.03	54.66–64.55	43.07–69.62
Inspection Time (ms)	55	66.92	45.39	59.50	34.00–85.00	17.00–255.00
Inspection Time (SWAY)	55	92.66	6.68	93.75	90.00–97.50	65.00–100.00
Working Memory (SWAY)	55	68.85	17.91	73.00	67.00–77.50	0.00–94.00
**Week 3**						
Reaction Time (ms)	55	270.38	48.70	265.50	230.00–300.50	197.50–396.50
Reaction Time (SWAY)	55	68.74	7.57	68.61	63.51–74.79	53.93–82.21
Impulse Control (ms)	55	345.50	71.50	324.00	295.50–391.50	248.00–584.50
Impulse Control (SWAY)	55	58.70	7.35	60.51	53.56–63.72	40.72–71.84
Inspection Time (ms)	55	61.35	33.67	51.00	34.00–85.00	17.00–187.00
Inspection Time (SWAY)	55	93.48	4.95	95.00	90.00–97.50	75.00–100.00
Working Memory (SWAY)	55	69.79	14.91	71.50	68.50–76.00	0.00–92.50

IQR, interquartile range; Md, median; ms, milliseconds; *n*, sample size; and SD, standard deviation.

**Table 2:** Friedman’s ANOVA Results.

	*Χ*^2^_F_	df	*p*
Reaction Time (ms)	1.98	2	0.37
Reaction Time (SWAY)	2.22	2	0.33
Impulse Control (ms)	2.09	2	0.35
Impulse Control (SWAY)	2.29	2	0.32
Inspection Time (ms)	0.94	2	0.62
Inspection Time (SWAY)	0.94	2	0.62
Working Memory (SWAY)	2.01	2	0.37

df, degrees of freedom; ms, milliseconds. Effect sizes were calculated as *r* values based on Wilcoxon signed-rank tests and were generally small (≤ 0.24).

**Table 3:** Test–Retest Reliability.

	ICC	95% CI	*F* test with true value 0			
		[lower, upper]	Value	df1	df2	*p*
**ICC for Weeks 1–3**							
Reaction Time (ms)	0.83	0.73, 0.89	5.71	54	108	<0.001
Reaction Time (SWAY)	0.83	0.73, 0.89	5.77	54	108	<0.001
Impulse Control (ms)	0.68	0.49, 0.80	3.09	54	108	<0.001
Impulse Control (SWAY)	0.80	0.68, 0.88	4.94	54	108	<0.001
Inspection Time (ms)	0.75	0.61, 0.85	3.98	54	108	<0.001
Inspection Time (SWAY)	0.75	0.61, 0.85	3.98	54	108	<0.001
Working Memory (SWAY)	0.88	0.81, 0.92	8.03	54	108	<0.001
**ICC for Weeks 1–2**							
Reaction Time (ms)	0.75	0.57, 0.85	3.96	54	54	<0.001
Reaction Time (SWAY)	0.74	0.56, 0.85	3.86	54	54	<0.001
Impulse Control (ms)	0.60	0.31, 0.76	2.48	54	54	<0.001
Impulse Control (SWAY)	0.76	0.60, 0.86	4.23	54	54	<0.001
Inspection Time (ms)	0.79	0.64, 0.88	4.72	54	54	<0.001
Inspection Time (SWAY)	0.79	0.64, 0.88	4.72	54	54	<0.001
Working Memory (SWAY)	0.83	0.72, 0.90	6.02	54	54	<0.001

Results of test–retest ICC Calculation for weeks 1–3 using mean-rating, absolute-agreement, and two-way mixed effects model and for weeks 1–2 using mean-rating, absolute-agreement, and two-way mixed effects model. CI, confidence interval; df, degrees of freedom; ICC, intraclass correlation coefficient; and ms, milliseconds.

The natural distributions of difference score data (Week 2–Week 1 and Week 3–Week 2) are presented in Tables 4, 5. ICCs (from Weeks 1 and 2) are presented in Table 3, and reliable change values, based on the reliable change CIs formula, are presented in Table 6. The reliable change values allow the reader to determine, with varying degrees of confidence, whether a particular level of improvement or decline in performance is within the range of measurement error. For example, for an examinee completing the Working Memory (SWAY) test across two testing sessions, an improvement of greater than about 11 points is unlikely to be due to measurement error at the 70% (liberal) confidence level, and an improvement of about 17 points is unlikely to be due to measurement error at the 90% (conservative) confidence level. For an examinee completing the Impulse Control (ms) test, a decline of about 77ms (slower reaction times) is unlikely to be due to measurement error at the 70% confidence level, and a decline of about 94ms is unlikely to be due to measurement error at the 80% confidence level. As seen in Table 7, we extracted several cutoff scores (from Tables 3, 6) for each test and then computed the percentages of the sample that scored below (worsened) or above (improved) those cutoff scores. These percentages were calculated from the frequency distributions of Week 2–Week 1 and Week 3–Week 2 difference scores.

**Table 4:** Interpreting change based on the natural distribution of Week 2–Week 1 difference scores.

	10%	15%	20%	Normal Variability	20%	15%	10%
Reaction Time (ms)	71.60	59.90	53.20	53.20−23.20	−23.20	−31.10	−41.70
Reaction Time (SWAY)	−11.93	−9.30	−8.33	−8.33–4.29	4.29	5.14	6.82
Impulse Control (ms)	57.40	43.60	36.90	36.90–−31.70	−31.70	−39.10	−49.00
Impulse Control (SWAY)	−7.19	−4.93	−4.37	−4.37–3.48	3.48	4.48	6.69
Inspection Time (ms)	54.40	42.50	25.50	25.50–−23.80	-23.80	−25.50	−37.40
Inspection Time (SWAY)	−8.00	−6.25	−3.75	−3.75–3.50	3.50	3.75	5.50
Working Memory (SWAY)	−9.40	−7.30	−6.00	−6.00–9.00	9.00	11.10	13.90

ms, milliseconds. The difference scores in this table were derived by subtracting the Week 1 scores from the Week 2 scores. The 10% score refers to the difference score that occurs in 10% or fewer of the total samples, and the 20% difference score refers to the difference score that occurs in 20% or fewer of the total sample. Normal variability represents the range of difference scores obtained by 60% of the sample. For example, a difference score of −41.70 on the Reaction Time (ms) test represents improved performance that is seen in 10% or fewer of the sample. A difference score of 36.90 on the Impulse Control (ms) test represents a decline in performance that is seen in 20% or fewer of the sample. For Working Memory (SWAY), Reaction Time (SWAY), Inspection Time (SWAY), and Impulse Control (SWAY), higher scores denote better performance. For Reaction Time (ms), Inspection Time (ms), and Impulse Control (ms), higher scores denote worse performance.

**Table 5:** Interpreting change based on the natural distribution of Week 3–Week 2 difference scores.

	10%	15%	20%	Normal Variability	20%	15%	10%
Reaction Time (ms)	43.70	33.80	28.60	28.60 – −29.30	−29.30	−0.49.60	−71.20
Reaction Time (SWAY)	−6.87	−5.54	−4.34	−4.34 – 4.23	4.23	5.19	9.55
Impulse Control (ms)	62.10	52.30	36.10	36.10 – −35.90	−35.90	−46.70	−54.00
Impulse Control (SWAY)	−6.65	−5.19	−4.13	−4.13 – 4.50	4.50	5.59	6.04
Inspection Time (ms)	28.90	22.10	17.00	17.00 – −25.50	−25.50	−34.00	−51.00
Inspection Time (SWAY)	−4.25	−3.25	−2.50	−2.50–3.75	3.75	5.00	7.50
Working Memory (SWAY)	−9.00	−6.90	−5.70	−5.70–6.90	6.90	9.30	13.20

ms, milliseconds. The difference scores in this table were derived by subtracting the Week 3 scores from the Week 2 scores. The 10% score refers to the difference score that occurs in 10% or fewer of the total samples, and the 20% difference score refers to the difference score that occurs in 20% or fewer of the total sample. Normal variability represents the range of difference scores obtained by 60% of the sample. For Working Memory (SWAY), Reaction Time (SWAY), Inspection Time (SWAY), and Impulse Control (SWAY), higher scores denote better performance. For Reaction Time (ms), Inspection Time (ms), and Impulse Control (ms), higher scores denote worse performance.

**Table 6:** Reliable change for seven SWAY indices based on weeks 1–2 ICCs.

	*M* (*SD*1)	*M* (*SD*2)	ICC	SEM1	SEM2	SE_diff_	0.70 CI	0.80 CI	0.90 CI
Reaction Time (ms)	261.19 (48.79)	274.33 (54.17)	0.75	24.40	27.09	36.46	±37.92	±46.67	±59.79
Reaction Time (SWAY)	70.16 (7.47)	68.24 (7.76)	0.74	3.81	3.96	5.50	±5.72	±7.04	±9.02
Impulse Control (ms)	352.08 (98.84)	343.08 (61.62)	0.60	62.51	38.97	73.66	±76.61	±94.28	±120.80
Impulse Control (SWAY)	57.93 (8.23)	58.69 (6.66)	0.76	4.03	3.26	5.18	±5.39	±6.63	±8.50
Inspection Time (ms)	61.51 (35.76)	66.92 (45.39)	0.79	16.39	20.80	26.48	±27.54	±33.89	±43.43
Inspection Time (SWAY)	93.45 (5.26)	92.66 (6.68)	0.79	2.41	3.06	3.90	±4.06	±4.99	±6.40
Working Memory (SWAY)	66.97 (17.84)	68.85 (17.91)	0.83	7.36	7.38	10.42	±10.84	±13.34	±17.09

CI, confidence interval; ICC, intraclass correlation coefficient; ms, milliseconds; SD, standard deviation; SE_diff_, SE of the difference; and SEM, standard error of measurement.

**Table 7:** Cutoffs for interpreting change.

		Percentage Worsened		Percentage Improved	
	Cutoff score	T1-T2	T2-T3	T1-T2	T2-T3
Reaction Time (SWAY Score)	5	27.3	16.4	14.5	14.5
	7	21.8	9.1	5.5	9.1
	8	20.0	5.5	3.6	7.3
	9	16.4	3.6	1.8	7.3
	10	12.7	3.6	1.8	7.3
	11	10.9	3.6	1.8	7.3
Impulse Control (SWAY Score)	5	12.7	14.5	10.9	14.5
	6	12.7	10.9	10.9	10.9
	7	10.9	7.3	5.5	3.6
Inspection Time (SWAY Score)	4	18.2	9.1	9.1	12.7
	5	18.2	9.1	9.1	12.7
	6	18.2	7.3	7.3	10.9
Working Memory (SWAY Score)	7	14.5	14.5	23.6	18.2
	10	9.1	7.3	14.5	10.9
	11	7.3	5.5	12.7	9.1
	13	5.5	5.5	9.1	7.3

T1, Time 1, T2, Time 2, and T3, Time 3. The percentages of the sample scoring at or below (worsened) or above (improved) the cutoffs are provided.

## Discussion

The current study provides a preliminary examination of test–retest reliability and reliable change estimates in four novel MCTs, assessing reaction time, impulse control, timed visual processing, and working memory in 55 community-dwelling adults. There were no statistically significant practice effects on the four MCTs. The test–retest reliability coefficients were adequate or better. Practical information relating to the interpretation of change on these tests is provided in Tables 4–7. The data provided in these tables allow the reader, on a preliminary basis, to determine whether or not particular scores are statistically reliable at different levels of certainty. That is, if an examinee does not improve or decline to a greater degree than is reflected in the confidence bands, the change in that person’s performance may be attributable to measurement error, or normal variability, and the probability that the change is clinically meaningful is reduced. When examining Tables 4–7, it can be seen that a change of five or more points is relatively uncommon for both the Reaction Time score and the Impulse Control score, although the Reaction Time score has more variability. A change of four or more points is relatively uncommon for the Inspection Time score. Worsening by seven or more points, or improving by 10 or more points, is relatively uncommon for the Working Memory score. Of course, in individual cases, clinical judgment is necessary to determine whether or not a particular improvement or decline is meaningful for that examinee. Overall, results from the current study provide preliminary support for clinical scientists to begin considering the SWAY MCTs as outcome measures in a variety of research settings. More studies are still needed, however, to establish more definitive recommendations for how to interpret change on these four SWAY MCTs.

### Psychometric Support for MCTs

Although scientific interest in MCTs is growing (Allard et al., 2014; Moore et al., 2017a; Sliwinski et al., 2018; Koo and Vizer, 2019; Weizenbaum et al., 2020), there is a dearth of psychometric data supporting these instruments (Charalambous et al., 2020), particularly with respect to test–retest reliability and reliable change. This leaves researchers with few options if they want to incorporate MCTs into their study designs.

Results of Brouillette et al. (2013) supported the test–retest reliability in one MCT measuring processing speed, and Timmers et al. (2014) found high test–retest correlations in an MCT assessing short term memory. Other investigations have reported good convergent and discriminant validity of MCTs compared to laboratory tests, with more shared variance between tests of similar constructs than between tests of distinct constructs (Moore et al., 2017a, 2020; Dupuy et al., 2018; Sliwinski et al., 2018). However, no studies to our knowledge have reported on estimates of reliable change in MCTs, and this is a limitation of the current literature, given that a major advantage of MCTs is their brevity and potential for repeatability (Allard et al., 2014; Sliwinski et al., 2018).

### Scientific Utility of MCTs

Research opportunities incorporating MCTs include delivering compensatory cognitive training in a telehealth format (e.g., Lawson et al., 2020), assessing the cognitive impact of pharmacotherapy for epilepsy (e.g., Eddy et al., 2011; Adams et al., 2017), measuring cognitive improvement following mindfulness-based treatment for ADHD (Poissant et al., 2019), and measuring long-term cognitive trajectories after mild traumatic brain injury and concussion (McAllister and McCrea, 2017), among others. In each of these cases, replacing traditional paper–pencil neuropsychological tests with remotely delivered MCTs would greatly reduce the time burden and overall cost associated with running the study. Alternatively, augmenting a conventional neuropsychological battery with MCTs would allow for both repeated, real-world assessments of cognition in context (MCTs), and standard evaluations of optimal cognitive performance in the laboratory (traditional testing), possibly reducing the likelihood of Type II (false negative) errors and providing a more complete picture of participants’ cognitive functioning.

### Limitations

Although promising, the current study has several limitations. First, important demographic data such as participants’ level of education, race, or ethnicity were not recorded, and, therefore, external validity is reduced. Relatedly, SWAY requires a smart phone with either an iOS version of 9.3 or higher, or an Android version 7.0 or higher, so the MCTs in this study are not accessible to everyone. Second, our sample is comprised primarily of younger adults (age: Md=23.0, IQR=20–30), so generalization to other age groups is constrained. Third, our sample is relatively small (*N*=55), which limits the robustness of the results. Fourth, our four MCTs assessed only a limited number of cognitive constructs (simple reaction time, response inhibition, timed visual processing, and working memory). That is, the tests do not represent a comprehensive cognitive assessment, and important domains such as delayed memory, verbal fluency, and set shifting were not measured. Fifth, although the ICCs for the time and SWAY scores are the same for the Reaction Time and Inspection Time tests, the ICCs for the Impulse Control SWAY scores are higher than the Impulse Control time scores. The reason for this is not clear, but it is presumably related to how the SWAY scores for Impulse Control are calculated. Relatedly, the Week 3–Week 2 difference scores for Reaction Time and Inspection Time (Table 5) do not align precisely with the Week 2–Week 1 difference scores for those tests (Table 4). We are not certain as to why these scores vary across Week 3–Week 2 compared to Week 2–Week 1, but it could be related to our relatively modest sample size in that a small number of people with more variable scores could influence these CIs. Sixth, the current study took place in a remote, supervised setting (by design). This context is less controlled and structured than conventional in-person, laboratory-based neuropsychological testing, while also being less ecologically valid than unsupervised remote testing (which is how MCTs are often examined). In other words, it lies somewhere between laboratory-based neuropsychological testing and unsupervised remote MCT with respect to the level of control and structure vs. ecological validity. Consequently, any inferences made from the current study results should account for the uniqueness of the setting. Seventh, our test–retest interval was only 1week. This brief interval is not representative of typical clinical practice in neuropsychology and may constrain the degree to which the test–retest reliability coefficients and reliable change indices generalize to other settings. Eighth, the current study presents reliability but not validity data for the SWAY MCTs. VanRavenhorst-Bell et al. (2021) reported preliminary support for the convergent validity of three of the current tests (Reaction Time, Impulse Control, and Visual Inspection) with the ImPACT Quick Test, but additional construct and criterion validity data are needed (e.g., convergent/discriminant validity with laboratory neuropsychological tests). Finally, although the current procedure is consistent with recommendations for administration of SWAY MCTs, other options are available. For example, a SWAY “Screening Test” involves the administration of each test one time, rather than two administrations being averaged together as was done in the current study. Our results should be generalized with caution outside of current study procedures.

### Conclusion

In summary, we report promising preliminary results with respect to test–retest reliability in four MCTs measuring reaction time, impulse control, timed visual processing, and working memory, in a sample of community-dwelling adults. Reliable change metrics allow for the estimation of statistically reliable improvements and declines in performance. However, the psychometric support for these MCTs remains limited, and more reliability and validity data are needed across the lifespan. Ultimately, following the accumulation of additional psychometric data, large, representative normative datasets, and research in clinical populations, we believe that these tests may be useful in clinical practice as an adjunct to traditional paper–pencil neuropsychological testing.

## Data Availability Statement

The statistical analyses and underlying data supporting the conclusions of this article will be made available by the authors to qualified researchers for research purposes, without undue reservation.

## Ethics Statement

The studies involving human participants were reviewed and approved by Wichita State University Institutional Review Board. The patients/participants provided their written informed consent to participate in this study.

## Author Contributions

RVP performed the literature review, helped conceptualize the statistical analyses, helped manage the database, conducted the statistical analyses, and wrote portions of the manuscript. GI conceptualized the study, helped with the literature review, helped conceptualize and interpret the statistical analyses, and wrote portions of the manuscript. MM collected the data and helped manage the database. HV-B supervised data collection and helped conceptualize the study. All authors critically reviewed drafts of the manuscript and contributed to the article and approved the submitted version

## Conflict of Interest

GI serves as a scientific advisor for Sway Medical, Inc. Operations, the company that created and distributes the tests used in this study. He also serves as a scientific advisor for Highmark, Inc., and NanoDx™ (formerly BioDirection, Inc.). He acknowledges unrestricted philanthropic support from ImPACT Applications, Inc., the Mooney-Reed Charitable Foundation, Boston Bolts, Heinz Family Foundation, National Rugby League, and the Spaulding Research Institute. He has received research funding from several test publishing companies [including ImPACT Applications, Inc., CNS Vital Signs, and Psychological Assessment Resources (PAR, Inc.)], the National Football League (NFL), and the NFL Players Association.

## Publisher’s Note

All claims expressed in this article are solely those of the authors and do not necessarily represent those of their affiliated organizations, or those of the publisher, the editors and the reviewers. Any product that may be evaluated in this article, or claim that may be made by its manufacturer, is not guaranteed or endorsed by the publisher.
